# Mitochondrial Parameters influence Chemosensitivity in Gastric Cancer Cells

**DOI:** 10.7150/jca.137748

**Published:** 2026-08-10

**Authors:** Kai-Chun Hong, Yi-Chun Chen, You-Syuan Lou, Chia-Chi Tsai, Xu-Chen Liu, Lei-Ni Liang, Chih-Cheng Cheng, Hsin-Hsien Yu, Bor-Chyuan Su

**Affiliations:** 1 School of Pharmacy, College of Pharmacy, Taipei Medical University, Taipei, Taiwan.; 2 School of Medicine, College of Medicine, Taipei Medical University, Taipei, Taiwan.; 3 Division of General Surgery, Department of Surgery, Wan Fang Hospital, Taipei Medical University, Taipei, Taiwan.; 4 Division of General Surgery, Department of Surgery, School of Medicine, College of Medicine, Taipei Medical University, Taipei, Taiwan.; 5 Department of Anatomy and Cell Biology, School of Medicine, College of Medicine, Taipei Medical University, Taipei, Taiwan.

**Keywords:** mitochondrial abundance, mitochondrial ATP, Bax, cytochrome c, UBR5, MAP1, chemosensitivity

## Abstract

Mitochondria play crucial roles in the survival of malignant cells in gastric and other types of cancers, and many cancer treatments act at least in part via mitochondrial effects. Although several measurable mitochondrial parameters have been associated with sensitivity of certain cancer cells to treatments, it remains unclear how these parameters differ among cancer cells according to differentiation level and histological type. It also remains unclear whether and how the effects of chemotherapy on mitochondrial parameters might be associated with sensitivity to treatment. In this study, we compared mitochondrial parameters in a panel of gastric cancer cell lines with different characteristics, including AGS (moderately differentiated), SNU-1 (poorly differentiated), KATO III (signet ring cell carcinoma; SRCC), and NUGC-4 (SRCC). We examined differences in basal mitochondrial parameters between the cell lines and the impacts of chemotherapy on each parameter. Our results showed that each cell line showed a different response pattern to cisplatin and 5-fluorouracil. For example, cisplatin was much more effective than 5-fluorouracil at killing SNU-1 gastric cancer cells, while KATO III cells exhibited minimal responses to both chemotherapies. These patterns of cytotoxicity were closely aligned with mitochondrial membrane potential. The different gastric cancer cell lines also displayed differences in basal mitochondrial parameters, but the basal measurements did not directly correlate with sensitivity to chemotherapy. Nevertheless, sensitivity was associated with an integrated profile of basal levels and effects of chemotherapy on mitochondrial parameters. Thus, our study highlights the association between mitochondrial parameters and chemosensitivity among gastric cancer cells with diverse characteristics. These findings may guide the development of more precise treatment strategies and targeted therapies for distinct types of gastric cancer.

## Introduction

Gastric cancer is the fifth most common cancer worldwide and the fourth leading cause of cancer-related deaths 1. Treatment options for gastric cancer are diverse and include surgery, chemotherapy, targeted therapy, and immunotherapy 1. Physicians choose the most appropriate treatment options based on the extent of tumor invasion and metastasis, as well as the presence or absence of specific molecular markers (human epidermal growth factor receptor 2; HER2) in the tumor 1. For example, the targeted drug trastuzumab (Herceptin) is often used to treat patients whose tumors are HER2-positive. Surgery is primarily used to remove non-metastatic tumors. However, gastric cancer patients are usually diagnosed with advanced stage disease involving distant metastasis, and these patients can only be treated with chemotherapy. Chemoresistance is a major obstacle in treating gastric cancer 2, and chemosensitivity is believed to be influenced by many factors, including Epstein-Barr virus (EBV) infection and chromosomal instability (CIN). As such, gastric cancer cells infected with EBV exhibit greater resistance to 5-fluorouracil (5-Fu) 3, while tumors with higher levels of CIN are more likely to develop resistance to chemotherapy 4. It remains controversial whether there is a correlation between gastric cancer cell sensitivity to chemotherapy and differentiation status. One study suggested that poorly differentiated gastric cancers are more sensitive to chemotherapy 5, but another indicated that patients with well-differentiated gastric cancers have better responses and prognosis to chemotherapy 6. Chemotherapy sensitivity in gastric cancer is also associated with histological subtype. For example, signet ring cell carcinoma (SRCC) is considered to be more resistant to chemotherapy and have poorer prognosis 7, 8. Furthermore, chemosensitivity of gastric cancer might be influenced by the expression levels of specific molecules, such as thymidylate synthase, excision repair complementation group 1, and glutathione S-transferase P1 9, 10.

While the previously mentioned factors and molecules influence chemosensitivity, several mitochondrial parameters are also closely linked to cell death. Nevertheless, the precise roles of these parameters in chemosensitivity remain controversial. Mitochondrial abundance is believed to regulate cell death, though the direction of effect may be context dependent. One study showed that higher mitochondrial abundance correlates with a greater ability of Jurkat cells to survive TRAIL stimulation, suggesting that higher mitochondrial abundance contributes to cell survival 11. However, another study argued that cells with greater mitochondrial abundance are likely more sensitive to TRAIL stimulation. In that study, more mitochondria were observed in dead cells than in living cells, and greater mitochondrial abundance was correlated with higher levels of pro-apoptotic proteins. Thus, it was concluded that cells with higher mitochondrial abundance are more sensitive to TRAIL 12. In addition, the main function of mitochondria is to produce ATP, and a previous study showed the intracellular ATP level is a critical determinant of chemotherapy sensitivity in colon cancer cells 13. In particular, reducing intracellular ATP enhanced the cytotoxic effects of 5-Fu and oxaliplatin 13. Along the same lines, it is known that the mitochondrial ATP synthase inhibitor oligomycin A potentiates the cytotoxicity of TRAIL 14 and menadione/ascorbate 15. Meanwhile, the opposite effect has also been observed, as oligomycin A prevented the cytotoxicity of nitric oxide 16 and tumor necrosis factor alpha 17. Nevertheless, the specific roles of mitochondrial abundance and mitochondrial ATP in gastric cancer chemosensitivity have not been thoroughly investigated.

The release of mitochondrial apoptotic proteins (e.g., cytochrome c) into the cytoplasm triggers activation of death-inducing signaling molecules, and many studies have shown that disrupting mitochondrial function can enhance cancer cell sensitivity to chemotherapy 18, 19. The release of mitochondrial apoptotic proteins is tightly regulated by numerous molecules. For example, the oligomerization of Bax on the mitochondrial membrane is a crucial step for the release of cytochrome c 20, 21. Bax is regulated by the ubiquitin protein ligase E3 component N-recognin 5 (UBR5) 20, 21, and this E3 ligase is believed to promote the degradation of the Bax activator modulator of apoptosis-1 (MAP1) to restrict apoptosis 21. In ovarian cancer studies, reduced UBR5 expression decreases the sensitivity of ovarian cancer cells to cisplatin (Cis) 21. Clinical studies have also shown that higher UBR5 expression correlates with larger tumor size, advanced TNM stage, and increased lymph node metastasis 22. Overall, elevated UBR5 expression is associated with poor prognosis in gastric cancer 22. However, the specific roles of MAP1 and UBR5 in chemosensitivity of gastric cancer remain unclear.

Although previous studies have investigated the roles of individual mitochondrial parameters in cancer chemosensitivity, it would be of benefit to conduct a comprehensive comparison of multiple mitochondrial characteristics among gastric cancer cells with different differentiation statuses and histological subtypes. Therefore, the aim of this study was to systematically evaluate the associations between chemosensitivity and multiple mitochondrial parameters in a panel of gastric cancer cell lines with distinct biological characteristics. Measured mitochondrial parameters included mitochondrial membrane potential, mitochondrial ROS, mitochondrial abundance, mitochondrial ATP, cytochrome c, Bax, MAP1, and UBR5.

## Materials and Methods

### Reagents

Propidium iodide (PI), 5-Fu, BioTracker ATP-Red, and RIPA buffer were purchased from MerckMillipore (Germany). Cis, 10-N-Nonyl acridine orange (NAO), mitoSOX red, and oligomycin A were purchased from MedChemExpress (USA). Enhanced three-color regular range protein markers (PM2510) was purchased from SMOBIO Technology (Hsinchu, Taiwan).

### Cell culture

AGS (moderately differentiated human gastric adenocarcinoma), SNU-1 (poorly differentiated human gastric carcinoma), and KATO III (human gastric SRCC) cell lines were purchased from Bioresource Collection and Research Center (Hsinchu, Taiwan). The NUGC-4 (human gastric SRCC) cell line was purchased from Riken BioResources Research Center (Japan). AGS, SNU-1, and NUGC-4 cells were maintained in RPMI 1640 (Thermo Fisher Scientific, USA) supplemented with Antibiotic-Antimycotic (Capricorn Scientific, Germany) and 10% fetal bovine serum (FBS; Cytiva, USA). KATO III cells were maintained in RPMI 1640 (Thermo Fisher Scientific, USA) supplemented with Antibiotic-Antimycotic (Capricorn Scientific, Germany) and 20% FBS (Cytiva, USA).

### Annexin V/PI double staining

After treatment, cells were collected and stained with annexin V-FITC (BioLegend) and PI for 15 min. Then, the fluorescence intensities of annexin V-FITC and PI were measured by flow cytometry (Beckman).

### Mitochondrial mass, mitochondrial membrane potential, mitochondrial ATP and mitochondrial ROS

In order to monitor mitochondrial volume, cells were stained with NAO (100 nM) for 30 min. Then, the cells were rinsed with PBS, and the fluorescence intensity of NAO was measured by flow cytometry. To assess mitochondrial membrane potential, cells were stained with TMRE (100 nM) for 15 min. Afterward, the cells were rinsed with PBS. The fluorescence intensity of TMRE was measured by flow cytometry. Mitochondrial ATP and mitochondrial ROS were labeled by BioTracker ATP-Red (10 μM) and mitoSOX red (500 nM), respectively. After labeling, cells were rinsed with PBS. The fluorescence intensities of BioTracker ATP-Red and mitoSOX red were measured by flow cytometry.

### Detection of Bax, cytochrome c, MAP1, and UBR5

Cells were first fixed with ice-cold methanol at -20°C for 15 min. Then, cells were fixed with 4% formaldehyde at room temperature for 15 min. Subsequently, the cells were blocked with 1% FBS in PBS at room temperature for 1 hour. Cells were then incubated overnight at 4 °C with Alexa Fluor 488-conjugated antibodies against Bax (1:200; BioLegend), cytochrome c (1:200; BioLegend), MAP1 (1:200; Santa Cruz Biotechnology) or UBR5 (1:200; Santa Cruz Biotechnology). After incubation, cells were washed three times with PBS, and the fluorescence intensities were measured by flow cytometry (Beckman Coulter). Debris was excluded based on forward scatter (FSC) and side scatter (SSC), and only intact cells were included in the analysis.

### Western blot analysis

Cell lysates were harvested in RIPA buffer. To compare samples, equal amounts of protein were separated on gradient SDS-PAGE gels and transferred onto PVDF membranes. The membranes were blocked with 1% Blotto and then incubated overnight at 4 °C on a shaker with the indicated primary antibodies. ATP5A1 (1:2,000 in 1% Blotto), ATP5B (1:4,000 in 1% Blotto), SOD2 (1:4,000 in 1% Blotto), and β-actin (1:20,000 in 1% Blotto) antibodies were purchased from Cell Signaling Technology. The SOD2K (Lys68) antibody (1:2,000 in 1% Blotto) was purchased from Abcam. After incubation with the primary antibody, the membranes were washed three times with TBS-T and then incubated with the appropriate HRP-conjugated secondary antibodies. Protein bands were visualized using enhanced chemiluminescence (ECL), and band intensities were quantified using ImageJ software.

### Transmission electron microscopy

SNU-1 cells were treated with Cis and 5-Fu, and mitochondrial ultrastructure was observed using transmission electron microscopy (TEM). Samples were prepared as previously described 23. Sections were observed using a TEM H600 (Hitachi, Japan).

### Statistical analysis

Statistical analyses were performed using GraphPad Prism version 8 (GraphPad Software, San Diego, CA, USA). Data are presented as the mean ± standard error of the mean (SEM) from at least three independent experiments unless otherwise indicated. Statistical significance was determined using one-way analysis of variance (ANOVA) followed by Tukey's multiple comparisons test for comparisons among multiple groups. A value of *P* < 0.05 was considered statistically significant.

## Results

### Cytotoxic effects of Cis and 5-Fu on various gastric cancer cell lines

**Figure 1:**
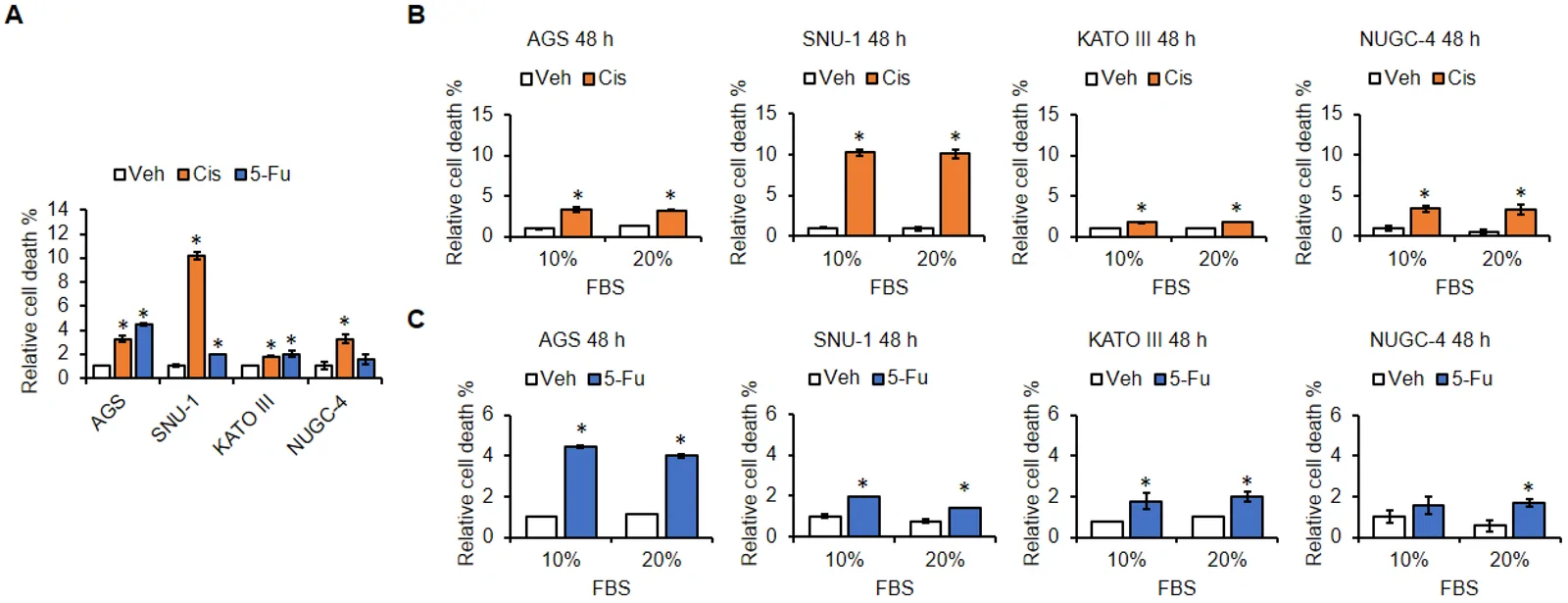
Cytotoxic effects of chemotherapy drugs on different types of gastric cancer cells. (**A**) Cells were treated with cisplatin (Cis; 20 μM) or 5-Fu (60 μM) for 48 h. Cytotoxicity was assessed by the annexin V/PI double staining assay. Cells were cultured in 10% or 20% FBS, then treated with Cis (20 μM) (**B**) or 5-Fu (60 μM) (**C**) for 48 h. Cell viability was assessed by the annexin V/PI double staining assay. *: *p* < 0.05. Data are presented as the mean ± SEM (n = 3 independent experiments). Statistical significance was determined by one-way ANOVA followed by Tukey's multiple comparisons test.

To investigate the chemosensitivity of different gastric cancer cells with varying degrees of differentiation and different histological types, AGS (moderately differentiated gastric cancer), SNU-1 (poorly differentiated gastric cancer), KATO III (SRCC), and NUGC-4 (SRCC) cell lines were treated with Cis and 5-Fu. Then, cytotoxicity was assessed by annexin V/PI double staining. We found that Cis generally caused greater cytotoxicity in gastric cancer cell lines compared to 5-Fu, with the sole exception of KATO III cells (Figure 1A). Among the four cell lines tested, 5-Fu demonstrated strong cytotoxicity only against AGS cells. It is important to note that SRCC gastric cancer is generally considered to have a poor response to chemotherapy 7, 8. However, only KATO III appeared to be less sensitive to chemotherapy, while the other tested SRCC line (NUGC-4) was not. Although both Cis and 5-Fu induced cell death in KATO III cells, the effects of both chemotherapies were relatively limited. The cytotoxic effect of Cis on NUGC-4 cells was comparable to its effect on AGS cells. These results suggest that not all SRCC gastric cancer cells respond uniformly to chemotherapy drugs. Of note, KATO III cells were cultured in medium containing 20% FBS, and high concentrations of FBS are known to influence the cytotoxic effects of cytotoxic agents 24. Thus, we sought to minimize the impacts of elevated FBS concentrations on chemosensitivity. Before administering Cis and 5-Fu, culture media were replaced by media containing 10% and 20% FBS, respectively (Figure 1B-C). The results for all cell lines showed that the serum concentration in the culture medium did not significantly affect the cytotoxic effects of the chemotherapeutic drugs. Therefore, the lower sensitivity to chemotherapy observed in KATO III cells is likely unrelated to the higher serum concentration used during culture.

### Effects of Cis and 5-Fu on the mitochondrial membrane potential and mitochondrial ROS

**Figure 2:**
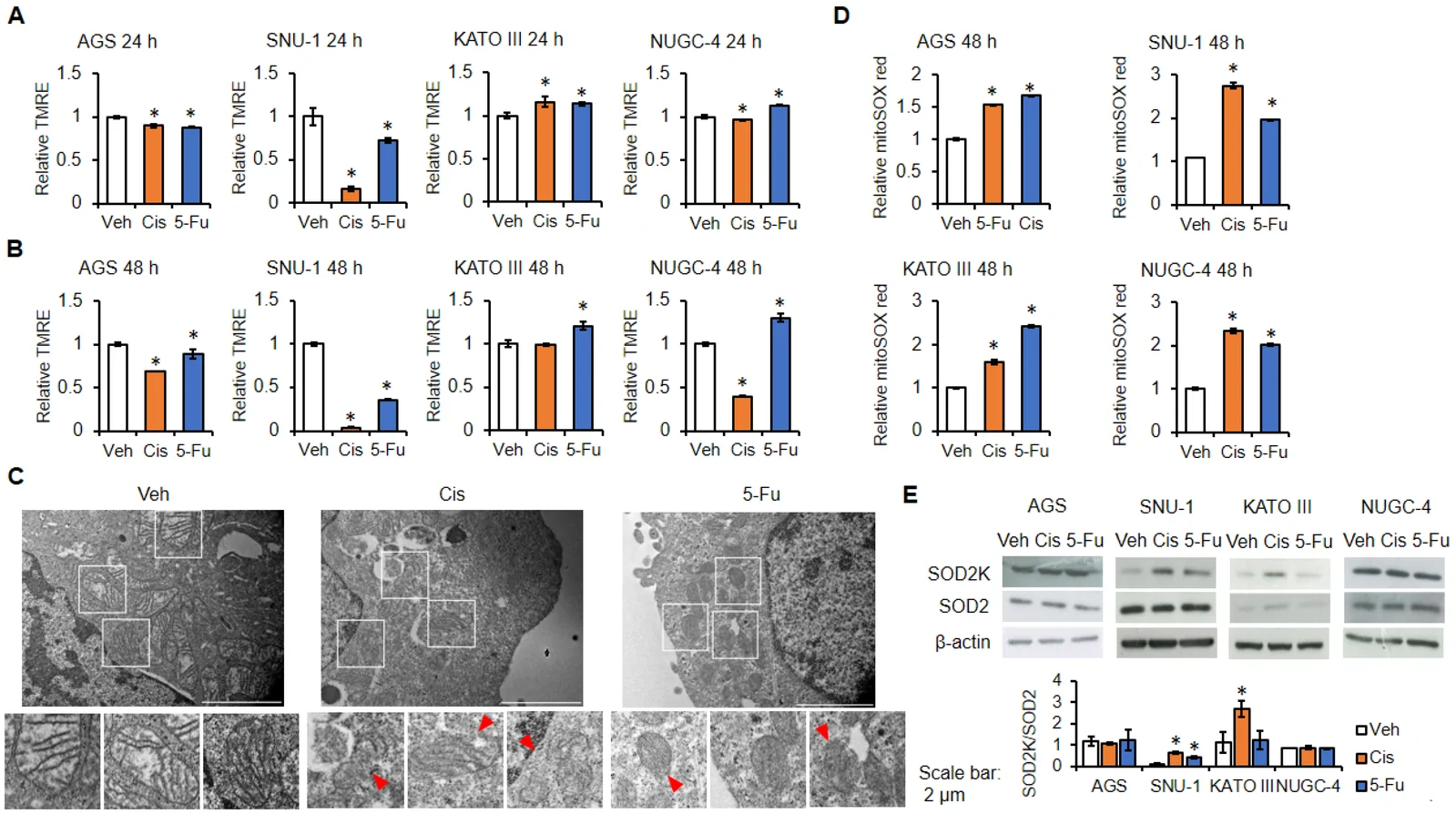
Effects of chemotherapeutic drugs on mitochondrial status in different gastric cancer cell lines. Cells were treated with cisplatin (Cis; 20 μM) or 5-Fu (60 μM) for 24 h (**A**) or 48 h (**B**); then, mitochondrial status was monitored by TMRE analysis. (**C**) SNU-1 cells were treated with cisplatin (Cis; 20 μM) or 5-Fu (60 μM) for 24 h, mitochondrial ultrastructures were observed by TEM. Red arrowheads indicate mitochondria with ruptured outer membranes. (**D**) Cells were treated with cisplatin (Cis; 20 μM) or 5-Fu (60 μM) for 48 h; then, mitochondrial ROS was monitored by mitoSOX red analysis. (**E**) Cells were treated with cisplatin (Cis; 20 μM) and 5-Fu (60 μM) for 24 h; then, levels of SOD2K and SOD2 were measured by western blot analysis. *: *p* < 0.05. Data are presented as the mean ± SEM (n = 3 independent experiments). Statistical significance was determined by one-way ANOVA followed by Tukey's multiple comparisons test.

We next compared the effects of chemotherapeutics on mitochondrial membrane potential in the four cell lines. Cells were incubated with Cis and 5-Fu for 24 h (Figure 2A) or 48 h (Figure 2B). TMRE was used to monitor mitochondrial membrane potential. We found that both Cis and 5-Fu altered mitochondrial membrane potential in all four cell lines. Notably, both Cis and 5-Fu decreased mitochondrial membrane potential in AGS and SNU-1 cells. However, in KATO III cells, both Cis and 5-Fu caused an increase in mitochondrial membrane potential. In NUGC-4 cells, Cis caused a decrease in mitochondrial membrane potential, while 5-Fu caused a slight increase. Compared with the other cell lines, SNU-1 cells showed particular sensitivity to Cis- and 5-Fu-mediated reductions in mitochondrial membrane potential. Therefore, we further performed TEM to examine the effects of Cis and 5-Fu on mitochondrial ultrastructure in SNU-1 cells. Treatment with either Cis or 5-Fu caused observable rupture of the outer mitochondrial membrane (Figure 2C). We next examined the effects of chemotherapeutic agents on mitochondrial ROS production in the four gastric cancer cell lines. As shown in Figure 2D, both Cis and 5-Fu increased mitochondrial ROS levels in all four cell lines. However, the extent of mitochondrial ROS accumulation differed depending on both the chemotherapeutic agent and the cell line. Specifically, Cis induced a greater increase in mitochondrial ROS than 5-Fu in SNU-1 and NUGC-4 cells, whereas 5-Fu elicited a greater ROS increase than Cis in KATO III cells. In AGS cells, Cis and 5-Fu induced comparable levels of mitochondrial ROS.

When comparing the effects of chemotherapeutic drugs on mitochondrial membrane potential (Figure 2B) and cytotoxicity (Figure 1A), we observed both similar and opposing trends. For example, in SNU-1 cells, Cis exhibited an obvious cytotoxic effect as well as a notable inhibitory effect on mitochondrial membrane potential. Cis also demonstrated relatively significant cytotoxic effects on AGS and NUGC-4 cells, which corresponded with a relatively strong inhibitory effect on the mitochondrial membrane potential in these cell lines. Meanwhile, 5-Fu showed a pronounced cytotoxic effect on AGS cells, but its impact on the mitochondrial membrane potential was comparatively weak. These findings suggest that while chemotherapeutic drugs commonly influence the mitochondrial membrane potential in various gastric cancer cell lines, this effect may not be the primary factor driving cytotoxicity in every cell line.

Building on these findings, we next sought to investigate the effects of chemotherapeutic agents (Cis and 5-Fu) on mitochondrial ROS levels in the four cell lines. Both Cis and 5-Fu increased mitochondrial ROS in all tested cell lines. Since mitochondrial ROS can be regulated by acetylation of mitochondrial superoxide dismutase (SOD2), we measured the effects of chemotherapeutic agents (Cis and 5-Fu) on the levels of SOD2 acetylation at lysine 68 in the gastric cancer cell lines (Figure 2E). Acetylation of SOD2 at lysine 68 leads to a loss of mitochondrial ROS scavenging function along with an increase in peroxidase activity 25. In AGS and NUGC-4 cells, Cis and 5-Fu had no effects on SOD2 acetylation (Figure 2E). However, in SNU-1 cells, both Cis and 5-Fu increased SOD2 acetylation. In KATO III cells, only Cis enhanced SOD2 acetylation. Interestingly, when we compared the effects of chemotherapeutic drugs on SOD2 acetylation and mitochondrial ROS levels across various cell lines, we found a positive correlation only in SNU-1 cells. In this line, Cis induced greater SOD2 acetylation and generated higher levels of mitochondrial ROS compared to 5-Fu. However, similar correlations were not observed in the other tested cell lines. These results indicate that chemotherapeutic drugs do not alter mitochondrial ROS levels by regulating SOD2 acetylation in all tested cell lines.

### Effects of chemotherapeutic agents on cytochrome C, MAP1, Bax and UBR5

**Figure 3:**
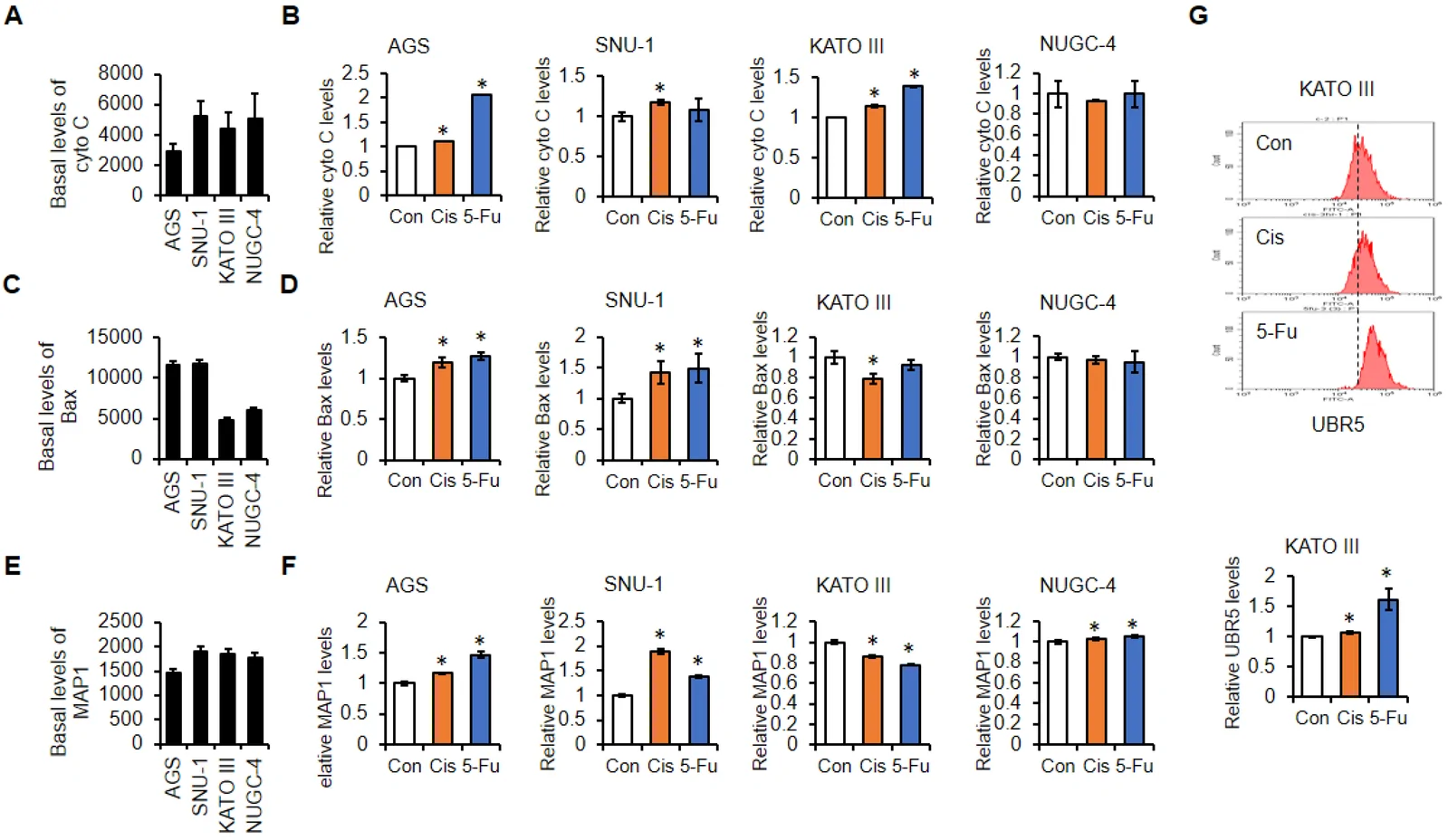
Effects of chemotherapeutic drugs on mitochondrial apoptosis-related proteins in different gastric cancer cell lines. (**A**) Basal cytochrome c (cyto c) levels in each cell line. (**B**) Cells were treated with cisplatin (Cis; 20 μM) or 5-Fu (60 μM) for 6 h; then, levels of cytochrome c were measured by flow cytometry. (**C**) Basal levels of Bax in each cell line. (**D**) Cells were treated with cisplatin (Cis; 20 μM) or 5-Fu (60 μM) for 6 h; then, levels of Bax were measured by flow cytometry. (**E**) Basal levels of MAP1 in each cell line. (**F**) Cells were treated with cisplatin (Cis; 20 μM) or 5-Fu (60 μM) for 6 h; then, levels of MAP1 were measured by flow cytometry. (**G**) Cells were treated with cisplatin (Cis; 20 μM) or 5-Fu (60 μM) for 3 h; then, levels of UBR5 were measured by flow cytometry. *: *p* < 0.05. Data are presented as the mean ± SEM (n = 3 independent experiments). Statistical significance was determined using one-way ANOVA followed by Tukey's multiple comparisons test.

We next analyzed the effects of Cis and 5-Fu on mitochondrial apoptosis-related proteins, including cytochrome c, Bax, MAP1, and UBR5. The results showed that AGS cells had the lowest total cytochrome c levels among the four cell lines (Figure 3A). Furthermore, we found that Cis stimulation increased cytochrome c in AGS, SNU-1, and KATO III cells, while 5-Fu stimulation significantly increased cytochrome c in AGS and KATO III cells (Figure 3B). Since cytochrome c release is regulated by Bax, we next analyzed Bax expression. We found that both SRCC gastric cancer cell lines, KATO III and NUGC-4, exhibited significantly lower Bax levels than the other cells (Figure 3C). Moreover, Cis and 5-Fu treatments increased Bax only in AGS and SNU-1 cells, while Cis decreased Bax in KATO III cells (Figure 3D). We then examined MAP1, a protein responsible for activating Bax, and found that AGS cells had the lowest MAP1 levels among the four cell lines (Figure 3E). Interestingly, Cis and 5-Fu treatments increased MAP1 in all cell lines except KATO III, where both drugs decreased MAP1 levels (Figure 3F). Since MAP1 activity is inhibited by UBR5 21, 22, we further hypothesized that the decrease in MAP1 in KATO III cells following Cis and 5-Fu treatments might be due to UBR5 upregulation. Consistent with this idea, Cis and 5-Fu both increased UBR5 expression in KATO III cells (Figure 3G).

### Effects of Cis and 5-Fu on mitochondrial abundance

**Figure 4:**
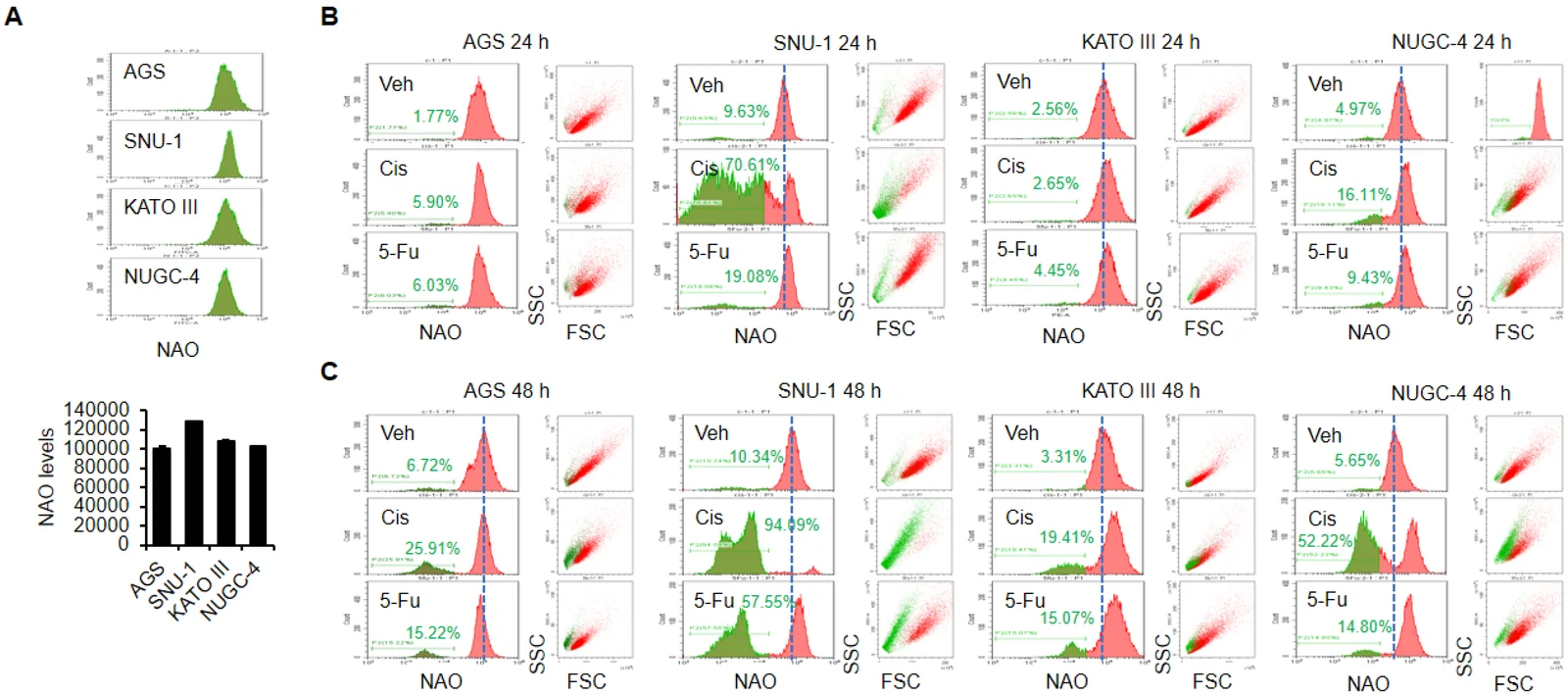
Effects of chemotherapeutic drugs on mitochondrial abundance in different gastric cancer cell lines. (**A**) Basal mitochondrial abundance in each cell line. (**B**) Cells were treated with cisplatin (Cis; 20 μM) or 5-Fu (60 μM) for 24 h, followed by measurement of mitochondrial abundance by flow cytometry. (**C**) Cells were treated with cisplatin (Cis; 20 μM) or 5-Fu (60 μM) for 48 h; then, mitochondrial abundance was measured by flow cytometry. Data are presented as the mean ± SEM (n = 3 independent experiments). Statistical significance was determined using one-way ANOVA followed by Tukey's multiple comparisons test.

We next evaluated mitochondrial abundance across the four cell lines and found that SNU-1 cells exhibited the highest levels (Figure 4A). In the other three cell lines, mitochondrial abundance was relatively similar. Next, we examined the effects of chemotherapeutic agents on mitochondrial abundance (Figure 4B-C). The effects became obvious only after 48 hours, except for the effect of Cis on SNU-1 cells. Treatments with Cis and 5-Fu consistently increased the proportions of gastric cancer cells with low mitochondrial abundance. Moreover, most of the cells with low mitochondrial abundance were presumably dead, as these cells were characterized by low FSC and high SSC in flow cytometry analyses. The increase in proportion of gastric cancer cells with low mitochondrial abundance induced by Cis paralleled the trend of Cis-induced cytotoxicity. Cis had the strongest effect on mitochondrial abundance in SNU-1 cells, while its effect on KATO III cells was the smallest. Compared to Cis, 5-Fu induced greater cytotoxicity in AGS cells, although its effect on reducing mitochondrial abundance was slightly less pronounced than that of Cis.

### Effects of Cis and 5-Fu on mitochondrial ATP

**Figure 5:**
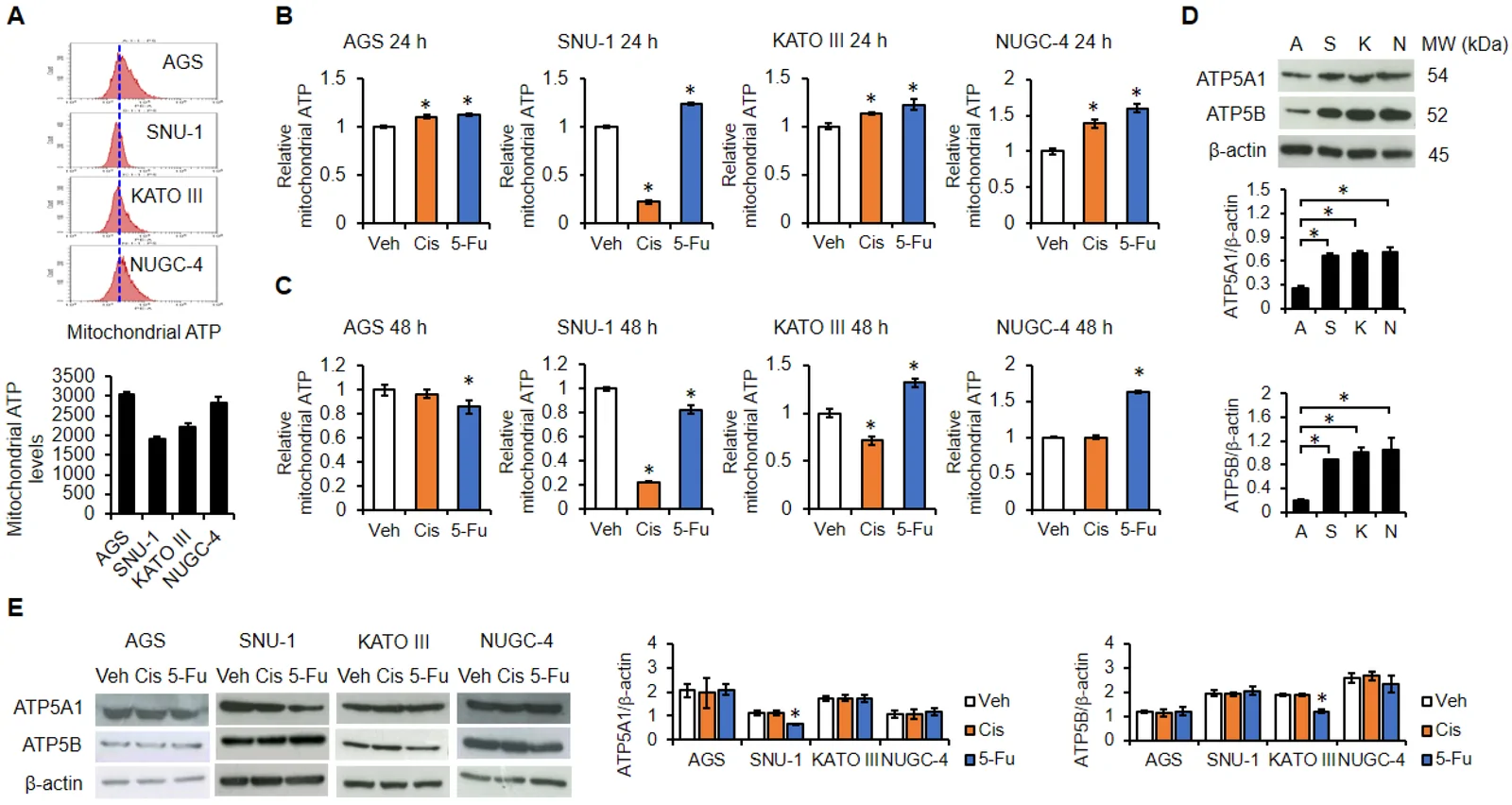
Effects of chemotherapeutic drugs on mitochondrial ATP in different gastric cancer cell lines. (**A**) Basal mitochondrial ATP levels in each cell line. (**B**) Cells were treated with cisplatin (Cis; 20 μM) or 5-Fu (60 μM) for 24 h; then, mitochondrial ATP levels were measured by flow cytometry. (**C**) Cells were treated with cisplatin (Cis; 20 μM) or 5-Fu (60 μM) for 48 h; then, mitochondrial ATP was measured by flow cytometry. (**D**) Basal levels of ATP5A1 and ATP5B in each cell line. (E) Cells were treated with cisplatin (Cis; 20 μM) or 5-Fu (60 μM) for 24 h; then, levels of ATP5A1 and ATP5B were measured by western blot analysis. *: *p* < 0.05. Data are presented as mean ± SEM from three independent experiments (n = 3 independent experiments). Statistical significance was determined by one-way ANOVA followed by Tukey's multiple comparisons test.

Since mitochondria are crucial sites of ATP synthesis, we analyzed the mitochondrial ATP levels in the four gastric cancer cell lines (Figure 5A). Among the cell lines, AGS cells had the highest mitochondrial ATP levels, while SNU-1 cells had the lowest. Each cell line was then treated with Cis and 5-Fu, and the alterations in mitochondrial ATP were measured (Figure 5B-C). We found that Cis and 5-Fu treatments generally increased mitochondrial ATP levels, except for Cis treatment of SNU-1 and KATO III cells. We also found that AGS cells exhibited the lowest levels of ATP-producing enzymes ATP5A1 and ATP5B among the four cell lines (Figure 5D). Furthermore, stimulation with either Cis or 5-Fu had limited effects on expression of these enzymes. The only observable effect was that 5-Fu slightly reduced ATP5A1 levels in SNU-1 cells and ATP5B levels in KATO III cells (Figure 5E).

## Discussion

Based on our observations, we can categorize the mitochondrial parameters evaluated in this study into two functional groups associated with cellular response to chemotherapeutic agents. The first functional group includes mitochondrial abundance and apoptosis-related proteins (cytochrome c, Bax, MAP1, and UBR5). Alterations in these parameters became more apparent after exposure to chemotherapeutic agents, suggesting that these factors may contribute to cellular responses following chemotherapy. It is likely that the orchestrated tuning of these factors modulates the sensitivity of different cell lines to chemotherapeutic agents. The second functional group is related to ATP-dependent drug resistance. Among the mitochondrial parameters evaluated in this study, basal mitochondrial ATP appeared to be one of the parameters most closely associated with chemotherapeutic drug sensitivity. Studies indicate that cancer cells with high ATP levels often exhibit multidrug-resistant phenotypes because mitochondrial ATP is essential for several key resistance mechanisms, including ABC transporter function 26 and maintenance of pro-survival signaling pathways in cancer cells 13. When cancer cells are exposed to chemotherapeutic drugs, sufficient mitochondrial ATP levels must be present for proper functioning of ABC transporters 26. These transporters can rapidly expel chemotherapeutic drugs from the cell, thereby reducing impacts on intracellular proteins or targets 26. Among the four cell lines tested, SNU-1 exhibited the lowest mitochondrial ATP levels (Figure 5A). Thus, it is possible that SNU-1 cells have relatively low ABC transporter-mediated drug efflux and higher intracellular drug accumulation 26. It is plausible that lower mitochondrial ATP levels may be associated with reduced ATP-dependent ABC transporter activity, although this possibility was not directly investigated in the present study. While variations in mitochondrial ATP levels may, at least in part, contribute to differences in chemosensitivity among gastric cancer cells, this hypothesis is based on previous literature and remains speculative, requiring further experimental validation.

Interestingly, Cis dramatically decreased mitochondrial ATP levels (Figure 5B-C) in SNU-1 cells, suggesting that the treatment may impair ABC transporter function. Additionally, SNU-1 cells showed the highest mitochondrial abundance (Figure 4A) and most elevated levels of mitochondrial apoptosis-related proteins, including cytochrome c (Figure 3A), Bax (Figure 3C) and MAP1 (Figure 3E). Cis stimulation further increased the expression of these apoptosis-related proteins (Figure 3B, D, F). Taken together, these findings could partially explain why Cis exerts such a strong inhibitory effect on mitochondrial membrane potential (Figure 2A-B) and cell viability in SNU-1 cells (Figure 1A). In contrast, 5-Fu exhibited a weaker and more variable effect on mitochondrial ATP levels in SNU-1 cells (Figure 5B-C), suggesting that the function of the ATP-driven ABC transporter is less affected. Therefore, it is possible that 5-Fu may be efficiently expelled from the cell. Moreover, 5-Fu increased the levels of Bax and MAP1 but did not elevate cytochrome c expression (Figure 3B, D, E). These differences likely account for the comparatively weaker cytotoxic effect of 5-Fu on SNU-1 cells relative to Cis (Figure 1A).

In contrast to SNU-1 cells, AGS cells contained the highest levels of mitochondrial ATP (Figure 5A), suggesting that this cell type may exhibit strong ABC transporter activity. Furthermore, both Cis and 5-Fu had relatively weak effects on mitochondrial ATP content in this cell line (Figure 5B-C). Although AGS cells exhibit the lowest basal levels of cytochrome c (Figure 3A) and MAP1 (Figure 3E), these cells expressed relatively high levels of Bax (Figure 3C). Additionally, 5-Fu was more effective than Cis at increasing the levels of all mitochondrial apoptosis-related proteins (Figure 3 B, D, E), which could partly explain why 5-Fu is more cytotoxic to AGS cells than Cis.

We further compared the results from KATO III and NUGC-4 cells, two SRCC gastric cancer cell lines. Between these lines, KATO III cells exhibited lower mitochondrial ATP content (Figure 5A). While the effect of Cis on mitochondrial ATP levels differed between lines, 5-Fu consistently increased mitochondrial ATP (Figure 5B-C). Further analysis of mitochondrial apoptosis-related proteins revealed that basal levels of cytochrome c and MAP1 in KATO III were comparable to the levels in SNU-1 and NUGC-4 cells (Figure 3A, E), but the KATO III cells exhibited the lowest levels of Bax (Figure 3C). Although both Cis and 5-Fu increased cytochrome c levels, treatment with Cis decreased Bax expression (Figure 3B, D). Furthermore, Cis and 5-Fu treatments also reduced the levels of MAP1 (Figure 3F), a Bax activator, while increasing the expression of UBR5 (Figure 3G), an E3 ubiquitin ligase responsible for degrading MAP1 21, 22. Previous studies have demonstrated that when MAP1 expression decreases, Bax activation is impaired, thereby inhibiting the efficient release of cytochrome c 21, 22. Overall, our findings are generally consistent with these previous reports, as chemotherapeutic treatment was associated with increased UBR5 expression and reduced MAP1 levels in KATO III cells. Collectively, our findings suggest that treatment of KATO III cells with chemotherapeutic agents, particularly 5-Fu, is associated with increased UBR5 expression and reduced MAP1/Bax-mediated apoptotic signaling. Previous literature suggests that responses may contribute to the relatively lower chemosensitivity observed in KATO III cells 20, 21.

NUGC-4 cells not only contain a relatively high basal level of mitochondrial ATP (Figure 5A), but Cis and 5-Fu further increase mitochondrial ATP levels (Figure 5B-C). Since elevated mitochondrial ATP levels may enhance the function of ABC transporters, we can hypothesize that Cis may accumulate to a cytotoxic intracellular concentration more readily than 5-Fu. Although Cis and 5-Fu do not further increase cytochrome c and Bax levels (Figure 3B, D), the treatments upregulate MAP1 expression (Figure 3F), which may sensitize Bax to activation and promote subsequent cytochrome c release. Higher intracellular concentrations of Cis and elevated MAP1 might partly explain why this chemotherapeutic has more pronounced effects on mitochondrial membrane potential and cell death compared to 5-Fu.

We also observed several other interesting relationships between the mitochondrial parameters we examined. Among the four tested cell lines, AGS exhibited the highest basal mitochondrial ATP levels (Figure 5A). However, this line also had the lowest expression levels of both ATP5A1 and ATP5B (Figure 5D). Furthermore, compared to the other three cell lines, AGS cells did not have the highest mitochondrial abundance (Figure 4A). These results suggest that mitochondrial ATP production is not solely influenced by the mitochondrial abundance and ATP synthases, but the level may also involve differences in cellular oxygen availability 27, electron transport chain efficiency 28, and/or mitochondrial calcium levels 29. Additionally, we noted that chemotherapeutic agents increased mitochondrial ROS in all four cell lines (Figure 2D). Previous studies have reported that chemotherapy-induced increases in mitochondrial ROS can play a critical role in promoting apoptosis in gastric cancer cells 28, 30. Consistent with these observations, both Cis and 5-Fu increased mitochondrial ROS levels in all four gastric cancer cell lines in the present study. However, the treatments did not robustly induce cell death in all cell lines (Figure 1A), suggesting that mitochondrial ROS accumulation is unlikely to be the sole determinant of chemosensitivity. The apparent discrepancy between ROS levels and cytotoxicity may indicate that differences in intracellular antioxidant defense systems or ROS tolerance mechanisms contribute to the distinct responses observed among gastric cancer cell lines. For example, variations in antioxidant pathways, including SOD2 activity, glutathione metabolism, catalase, and the NRF2 signaling pathway, may influence the ability of cancer cells to tolerate chemotherapy-induced mitochondrial ROS 31, 32. However, these mechanisms were not directly investigated in the present study and therefore remain speculative explanations based on previous literature 31, 32. In contrast, the extent to which chemotherapeutic agents alter mitochondrial membrane potential (Figure 2A-B) showed a consistent parallel with the cell death (Figure 1A). Overall, the four gastric cancer cell lines exhibited distinct mitochondrial characteristics and chemotherapy response patterns. Among these patterns, SNU-1 cells displayed the lowest basal mitochondrial ATP levels along with the greatest sensitivity to chemotherapeutic agents. In contrast, KATO III cells exhibited relatively lower chemosensitivity and a greater induction of UBR5 expression following chemotherapeutic treatment. AGS and NUGC-4 cells demonstrated intermediate mitochondrial profiles and chemotherapy responses. Collectively, these findings suggest that variations in chemosensitivity among gastric cancer cell lines are associated with multiple mitochondrial characteristics rather than a single mitochondrial parameter, highlighting the complexity of mitochondrial impacts on chemotherapy responses.

## Limitations

This study compared mitochondrial parameters and chemosensitivity in established human gastric cancer cell lines with different differentiation statuses and histological subtypes. However, cancer cell lines cannot fully recapitulate the biological heterogeneity and complexity of primary gastric tumors. In addition, no patient-derived samples or organoid models were included in the present study. Therefore, future studies using patient-derived tumor samples and gastric cancer organoids will be important to validate the associations observed in this study and to further assess their clinical relevance.

## Conclusions

Mitochondria are widely recognized as important intracellular targets of chemotherapeutic agents 33, 34. In this study, we systematically compared the effects of chemotherapeutic agents on multiple mitochondrial parameters in gastric cancer cell lines representing different differentiation statuses and histological subtypes. Our findings provide a comprehensive overview of the associations between mitochondrial characteristics and chemosensitivity among distinct gastric cancer cell types. Although further validation using patient-derived samples and organoid models is required, these findings provide a foundation for future studies aimed at identifying mitochondrial features associated with chemotherapy responses and may facilitate the development of more precise therapeutic strategies for gastric cancer.

## Data Availability

The datasets used and/or analyzed during the current study are available from the corresponding author on reasonable request.
